# Peripheral Blood Mononuclear Cells of Mycetoma Patients React Differently to *Madurella mycetomatis* Antigens than Healthy Endemic Controls

**DOI:** 10.1371/journal.pntd.0002081

**Published:** 2013-04-25

**Authors:** Ehab A. M. Elagab, Maowia M. Mukhtar, Ahmed H. Fahal, Wendy W. J. van de Sande

**Affiliations:** 1 Mycetoma Research Centre, University of Khartoum, Khartoum, Sudan; 2 Institute of Endemic Diseases, University of Khartoum, Khartoum, Sudan; 3 Erasmus Medical Center, Department of Medical Microbiology and Infectious Diseases, Rotterdam, the Netherlands; University of California San Diego School of Medicine, United States of America

Mycetoma is a chronic, specific, granulomatous progressive, and destructive inflammatory disease of mainly the foot caused by either fungi (eumycetoma) or bacteria (actinomycetoma) [1]. Mycetoma is endemic in many tropical and subtropical regions, and it prevails in what is known as the mycetoma belt, which stretches in bands between the latitudes of 15° South and 30° North of the equator. With massive international travel, it also has been occasionally reported from different temperate regions [1]. The triad of a painless subcutaneous mass, multiple sinus formation, and purulent or sero-purulent discharge that contains grains is characteristic of mycetoma. It usually spreads to involve the skin, deep structures, and bone-producing massive deformities. Due to these deformities, mycetoma has a high morbidity rate, with a huge impact on the patient, family, and the community [1].

Since mycetoma has been highly neglected, much is still not known regarding the susceptibility, resistance, infection route, disease progression, and response to medical treatment of mycetoma. Based on antibody measurements in earlier studies, it was demonstrated that although most people living in mycetoma-endemic areas are exposed to the causative agent, only a few developed mycetoma [2], [3]. Therefore, the question arises of why some people develop mycetoma and others not, and when mycetoma is developed, why some patients respond well to treatment and others not. The answer to these questions could lie in the different reactions toward *M. mycetomatis* by the immune system between individuals who develop mycetoma and individuals who do not.

So far there has been limited data on immune response toward mycetoma infection. The only data available were derived from animal models and actinomycetoma patients. From mouse models it was evident that mycetoma only occurred when *M. mycetomatis* was inoculated with an adjuvant (either soil or Freunds incomplete adjuvant), both predisposing toward a Th2 response [4], [5]. Remarkably, in 1974, Cavanagh demonstrated that *M. mycetomatis* emulsified in the Th1-predisposing Freunds complete adjuvant (FCA) could not induce mycetoma in L20 mice [6], [7]. Furthermore, when Peripheral Blood Mononuclear Cells (PBMCs) from actinomycetoma patients were stimulated with antigens from *Nocardia brasiliensis*, a Th2-type cytokine profile was detected [8]. This could indicate that the causative agent probably induces a Th2 response that facilitates further growth and the development of a clinically significant mycetoma lesion. In order to determine if a Th2 cytokine profile is indeed associated with mycetoma development and a Th1 cytokine profile with protection against mycetoma, this prospective, cross-sectional study was conducted at the Mycetoma Research Centre and the Institute of Endemic Diseases, University of Khartoum, Khartoum, Sudan, after obtaining ethical clearance from the Soba University Hospital Ethical Committee to determine the profile of four cytokines in both healthy endemic controls and mycetoma patients. The study included 27 patients with confirmed eumycetoma due to *Madurella mycetomatis* treated with 800 mg/d ketoconazole and 21 normal individuals from eumycetoma-endemic areas (Table 1). The present study was approved by the Ethics Committee of Soba University Hospital, Khartoum, Sudan. Written informed consents were obtained from the participants prior to their enrollment in the study. For each study, individual 10 ml of venous blood were collected in heparinized blood collection tubes. From the collected blood, peripheral mononuclear cells were isolated using Ficoll-Hypaque gradient centrifugation as described previously [9]. Stimulation of cells was done using 50 µl of uncharacterized culture filtrate of *M. mycetomatis*. The culture filtrate of *M. mycetomatis* was prepared by culturing *M. mycetomatis* for 3 wk at 37°C in RPMI-1640 medium supplemented with morpholinopropanesulfonic acid (MOPS) and phenol red and by filtrating this through a 0.45 micron pore size filter joined to a 20 ml syringe to purify any mycelia and debris. The filtered culture filtrate was stored at −20°C until used. To stimulate the isolated PBMCs, 1×10^6^ cells were plated into a 24-well flat-bottomed cell culture plate. Stimulation of PBMCs was done using 50 µl of culture filtrate of *M. mycetomatis*. For each patient and healthy control, some wells were left unstimulated to serve as control. The culture plates were incubated at 37°C with 5% CO2. After 72 h, the cells were harvested by centrifugation at 1,200 rpm for 3 min, and the supernatants were then collected for cytokine measurement by sandwich ELISAs using BD OptEIA ELISA Set B (Catalog No. 550534).

**Table 1:** Patient demographics.

		Eumycetoma Patients	Healthy Endemic Controls
Number (*n*)		27	21
Gender (male/female)		22/5	19/2
Mean age years (range)		23.5 (9–45)	33.9 (22–53)
Lesion site		Foot	NA
Lesion size (*n*)	Large	18	NA
	Medium	5	NA
	Small	4	NA
Ketoconazole responding (yes/no)		11/8	NA
Causative agent		*M. mycetomatis*	NA

NA, not applicable.

**Figure 1:**
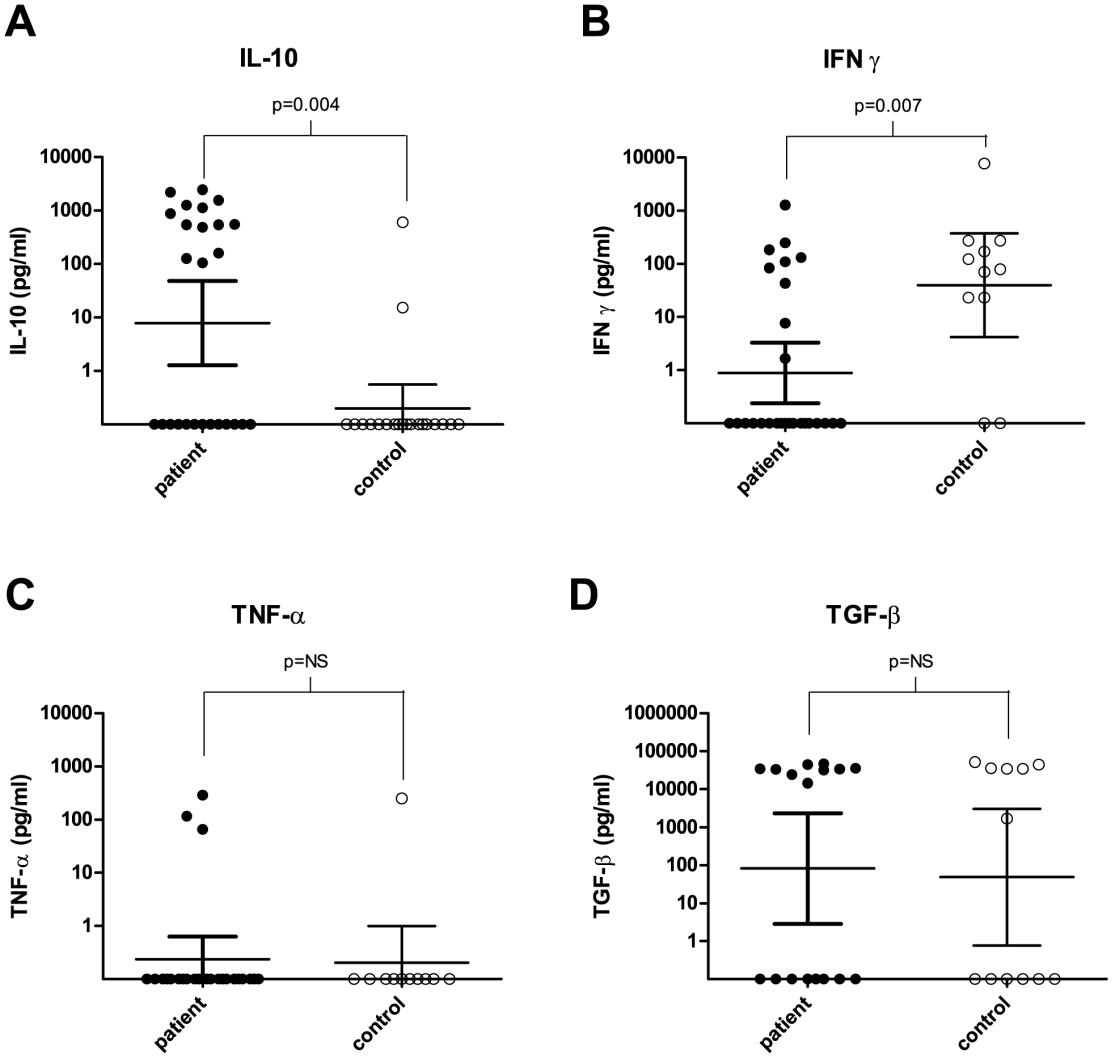
Cytokine concentrations of stimulated PBMCs of eumycetoma patients and healthy controls. (A) IL-10 concentrations (pg/ml) measured after stimulating PBMCs from eumycetoma patients and healthy endemic controls with *M. mycetomatis* antigen. Statistically significantly different concentrations were found between eumycetoma patients and healthy controls (*p* = 0.004) as determined by the Mann–Whitney *U* test. (B) IFN-γ concentrations (pg/ml) measured after stimulating PBMCs from eumycetoma patients and healthy endemic controls with *M. mycetomatis* antigen. Statistically significantly different concentrations were found between eumycetoma patients and healthy controls (*p* = 0.007) as determined by the Mann–Whitney *U* test. (C) TNF-α concentrations (pg/ml) measured after stimulating PBMCs from eumycetoma patients and healthy endemic controls with *M. mycetomatis* antigen. No statistically significantly different concentrations were found between eumycetoma patients and healthy controls (*p* = 0.85) as determined by the Mann–Whitney *U* test. (D) TGF-β concentrations (pg/ml) measured after stimulating PBMCs from eumycetoma patients and healthy endemic controls with *M. mycetomatis* antigen. No statistically significantly different concentrations were found between eumycetoma patients and healthy controls (*p* = 0.89) as determined by the Mann–Whitney *U* test.

After stimulating the PBMCs of both patients and healthy endemic controls with the *M. mycetomatis* antigen preparation, it was noted that the PBMCs of the patients reacted differently to this antigen than the healthy controls. Statistically significantly higher IL-10 concentrations were detected in all the patients (mean 458.2 pg/ml) compared to the healthy controls (mean 30.7 pg/ml) (Figure 1A, Mann–Whitney, *p* = 0.004, GraphPad Prism 5.01). In contrast, the INF*-γ* concentrations were significantly higher in the control population (mean 792.8 pg/ml) than in the patient group (mean 77.4 pg/ml) (Figure 1B, Mann–Whitney, *p* = 0.007). Generally it was noted that when PBMCs produced INF*-γ* upon stimulation with the antigen, no IL-10 was produced and vice versa. There were no significant differences in TNFα or TGF-β concentrations between patients and control groups (Figure 1C and 1D, Mann–Whitney, *p*>0.05). In order to determine if mycetoma patients with larger lesions had higher levels of IL-10 and lower levels of INF-*γ*, the IL-10 and INF-*γ* levels were stratified according to lesion size. Although in general higher concentrations of Il-10 and lower levels of INF-*γ* were found in the massive lesions, it appeared that this was not statistically different from the smaller lesion sizes (Kruskall–Wallis, *p*>0.05; GraphPad Prism 5.01). There is only one other study with which we could use to compare our results [10]. Castro–Matteotti used three different *Nocardia brasiliensis* extracts to stimulate PBMCs from seven actinomycetoma patients, seven tuberculosis patients, and seven healthy controls and measured the INF-*γ* response [10]. None of the extracts was able to stimulate INF-*γ* production in humans [10]. This is in contrast to our findings. When we stimulated the healthy controls in our study with a *M. mycetomatis* extract, the PBMCs of the healthy controls produced INF-*γ*. Clearly, the PBMC response toward eumycetoma antigens and actinomycetoma antigens differs.

At the moment, eumycetoma is treated with surgery and prolonged antifungal therapy with ketoconazole. Ketoconazole is known to suppress the production of Th2-type cytokines IL-4 and IL-5; therefore, the working mechanism of this drug in mycetoma might be 2-fold: inhibiting fungal growth and reversing the immune system toward a Th1 response [11], [12]. Unfortunately not all patients respond well to ketoconazole treatment. Many still show recurrent infections, even if the fungal species itself is susceptible toward this drug [13]. Furthermore, it also determined if patients responding to ketoconazole had different cytokine profiles than patients not responding to this drug. All patients were treated with 800 mg/d of ketoconazole for at least 6 mo. If a patient was responding to this treatment, the size of the lesion decreased, discharging sinuses closed, and there was a noted pain relief. According to these criteria, the patients were further divided into two groups: patients who responded to 800 mg/d ketoconazole (*n* = 11) and those who did not respond (*n* = 8). PBMCs of patients who responded to ketoconazole therapy produced similar levels of IL-10, INF-γ, TNF-α, or TGF-β as patients who did not respond to this therapy (Mann–Whitney, *p* = *ns* for all cytokines).

In conclusion, this study showed that mycetoma patients produce high concentrations of IL-10 that correlate with the disease severity. IFN-γ seems to be associated with protection against mycetoma, and it may indicate past subclinical infections. The four cytokine concentration levels were not affected by a medical treatment response. An important limitation of the present study is the small number of patients included. Further studies at the field level in endemic areas and on controls from nonendemic areas that study cytokine profile in serum and in the histopathological lesion are recommended for more in-depth understanding of the immune responses of the mycetoma patients.
